# Mutational Analysis of the Terminal Protein Tpg of *Streptomyces* Chromosomes: Identification of the Deoxynucleotidylation Site

**DOI:** 10.1371/journal.pone.0056322

**Published:** 2013-02-14

**Authors:** Chien-Chin Yang, We-Chi Sun, Wan-Yu Wang, Chi-Hung Huang, Fang-Shy Lu, Shu-Min Tseng, Carton W. Chen

**Affiliations:** 1 Department of Chemistry, Chung-Yuan Christian University, Chung-li, Taiwan; 2 Institute of Biotechnology, National Taipei University of Technology, Taipei, Taiwan; 3 Department of Life Sciences, Institute of Genome Sciences, National Yang-Ming University, Shih-Pai, Taipei, Taiwan; King’s College, London, United Kingdom

## Abstract

The linear chromosomes and linear plasmids of *Streptomyces* are capped by terminal proteins (TPs) covalently bound to the 5′ ends of the DNA. The TPs serve as primers for DNA synthesis that patches in the single-stranded gaps at the telomeres resulting from the bi-directional replication (‘end patching’). Typical *Streptomyces* TPs, designated Tpgs, are conserved in sequence and size (about 185 amino acids), and contain a predicted helix-turn-helix domain and a functional nuclear localization signal. The Tpg-encoding gene (*tpg*) is often accompanied by an upstream gene *tap* that encodes an essential telomere-associating protein. Five lone *tpg* variants (not accompanied by *tap*) from various *Streptomyces* species were tested, and three were found to be pseudogenes. The lone *tpg* variant on the SLP2 plasmid, although functional, still requires the presence of *tap* on the chromosome for end patching. Using a combination of *in vitro* deoxynucleotidylation, physical localization, and genetic analysis, we identified the threonine at position 114 (T114) in Tpg of *Streptomyces lividans* chromosome as the deoxynucleotidylated site. Interestingly, T114 could be substituted by a serine without destroying the priming activity of Tpg *in vitro* and *in vivo*. Such T114S substitution is seen in and a number of pseudogenes as well as functional Tpgs. T114 lies in a predicted coil flanked by two short helixes in a highly hydrophilic region. The location and structural arrangement of the deoxynucleotidylated site in Tpg is similar to those in the TPs of phage ø 29 and adenoviruses. However, these TPs are distinct in their sequences and sizes, indicating that they have evolved independently during evolution. Using naturally occurring and artificially created *tpg* variants, we further identified several amino acid residues in the N-terminus and the helix-turn-helix domain that were important for functionality.

## Introduction

Soil bacteria of the genus *Streptomyces* possess linear chromosomes and linear plasmids that are capped by terminal proteins (TPs) covalently bound to the 5′ ends of the DNA [1], [2]. Replication of these linear replicons is accomplished in two steps: (*i*) bidirectional replication initiated from an internal origin, which results in single-stranded gaps at the 3′ end; and (*ii*) patching of the single-stranded gaps by TP-primed DNA synthesis [3]. The TPs remain covalently attached to the telomeres of the linear *Streptomyces* replicons after replication.

The patching TP-primed DNA synthesis in *Streptomyces* differs from the replicative TP-primed synthesis involved in the replication of adenoviruses [4] and phage ø29 [5], which have been extensively studied. In end patching, only about 300 nt of single-stranded gaps need to be filled [3], whereas replicative TP-primed synthesis duplicates the whole replicons end-to-end.

In *Streptomyces*, most TPs are highly conserved in sequences and size (about 185 amino acids). These conserved TPs are designated archetypal TPs, and are encoded by *tpg* gene, which typically lie downstream of a *tap* gene in the same operon [6], [7]. Tap is also essential for replication of linear *Streptomyces* replicons. It binds specifically to a secondary structure formed by the single-stranded 3′ overhang during replication, and presumably recruits Tpg to the telomere location for the end patching reaction [8].

The *tap-tpg* operon generally lies in the terminal region of the *Streptomyces* chromosomes. A few linear plasmids also contain the *tap-tpg* operon or a lone *tpg* homolog. In addition, apparent *tpg* pseudogenes are found in sequenced *Streptomyces* genomes. The first lone *tpg* homolog was discovered in the 15.4-kb terminal sequences shared by the *S. lividans* chromosome and the right end of SLP2 [9]. This homolog, designated *tpg* ^SLP2.38^, exhibited atypical codon usage and encoded a product (183-amino acid) with a number of deletions and insertions. It was presumably a pseudogene. Subsequently, more putative *tpg* pseudogenes have been found in the linear plasmids and chromosomes in many *Streptomyces species*, for example, *S. avermitilis* [10], *S. ambofaciens* [11], *S. griseus* [12], [13], and *S. violaceoruber* [14].

The N-terminal halves of Tpgs contain a number of predicted functional and structural domains [7]: (*i*) a helix-turn-helix (HTH) domain overlapping a segment similar to part of the thumb domain of HIV reverse transcriptase; (*ii*) a mono-partite nuclear localization signal (NLS) that is functional in targeting nuclei of human [15] and plant [16] cells, but is not essential for end patching [15]; (*iii*) an amphiphilic ß-strand that may be involved in protein-protein interactions or protein-membrane interactions. Interactions between the *Streptomyces* telomeres *in vivo* have been recently demonstrated [17].

SCP1, a 350-kb linear plasmid in *Streptomyces coelicolor* A3(2), encodes its own TP, Tpc [18]. Tpc shares no homology with Tpgs, and is significantly larger (259 amino acids). The coding gene, *tpc,* is also downstream from a gene *tac* that is essential for replication of SCP1. Tpc, like Tpgs, contains an HTH domain in the N-terminal region and a functional bi-partite NLS in the central region [15].

Recently, Yang *et al*. [19] demonstrated *in vitro* deoxynucleotidylation of Tpg^Sli^ of *S. lividans*, in which dCMP, the first nucleotide at the 5′ ends of the *S. lividans* chromosome, is specifically covalently attached to Tpg^Sli^ in the *in vitro* reactions. The dCMP was attached to a Thr residue of Tpg^Sli^. There are 11 Thr residues in Tpg^Sli^. In comparison, the adenovirus [20] and ø29 [21] DNAs are attached to a Ser residue on their TPs. Other than these, little is known about the mechanism of end patching in replication of linear *Streptomyces* replicons.

In this study, combining biochemical and genetic approaches, we identified T114 at the C-terminus of Tpg^Sli^ as the attachment site of the telomere DNA. Interestingly, this residue may be substituted by a Ser without destroying the deoxynucleotidylation function of Tpg *in vitro* and the priming function *in vivo*. Examination of five lone *tpg* homologs identified three of them to be pseudogenes. Interestingly all these pseudogenes have the T114S substitution, and therefore the defect must lie somewhere else. Moreover, we identified several amino acid residues in the neighborhood of T114, the N-terminus, and the helix-turn-helix (HTH) domain of Tpg^Sli^ that were important for the functionality of Tpg^Sli^.

## Materials and Methods

### Bacterial Cultures and Molecular Manipulations

Genetic manipulations of *E. coli* and *Streptomyces* were performed according to the methods of Kieser et al. [22]. *E. coli* BL21(DE52) (Stratagene) and *S. lividans* MR04 [23] were used for expression of protein. pLUS980 plasmid containing a 4.5-kb *Bgl*II fragment spanning the *tap-tpg* operon from *S. coelicolor* cosmid 8D11 [24] was obtained from Chia-Hui Ke.

### Protein Expression in *E. coli* BL21(DE52) and Purification

All the proteins used here were constructed in vector pet-15b (Novagen) where His6 is tagged to the N-terminal, or in pet-22b to the C-terminal with the purpose of purification, or vector pRSET A (Invitrogen) without His6-tagging. Protein expression was induced by IPTG (isopropyl-b-D-1-thiogalactopyranoside). The cells were centrifuged and sonicated in A8 buffer (50 mM TrisHCl pH8, 20 mM NaCl, 10% glycerol) and the His6-tagged protein purified using nickel-bound resin (Bioman) according to the protocols provided by the manufacturer. Insoluble His6-tagged proteins were purified in denatured state using nickel-bound resin and eluted in Elution buffer containing 6 M urea, refolded in Elution buffer by step-wise dialysis, and stored at −20°C in Elution buffer containing 5% DMSO (dimethyl sulfoxide). The concentrations of proteins were determined by the Bradford method [25]. Tpg without the His6-tag was purified by electrophoresis in 12% SDS-PAGE as described previously [19].

### In vitro Deoxynucleotidylation and Purification of the Labeled Tpg

*In vitro* deoxynucleotidylation of untagged Tpg in cell extracts with alpha-[^32^P]-dCMP was based on the procedure described previously [19]. The labeled proteins were purified by SDS-PAGE or by immuno-precipitation using protein A-Sepharose and anti-Tpg^Sli^ antibody. With this procedure, Tpg with various lengths of deoxynucleotides were recovered.

An alternative procedure employed purified His6-Tpg^Sli^, His6-Tap^Sli^, and His6-tagged DinB1 DNA polymerase (product of gene SCO1380 of *S. coelicolor*). A 20-µl mixture, containing 0.3 µg each of His6-Tpg^Sli^, His6-Tap^Sli^, and His6-DinB1, 0.1 pmole of denatured 99-bp telomere DNA of *S. lividans* chromosome (prepared by PCR), 2.5 mM ATP, 10 mM Tris-HCl (pH7.5), 7 mM Mg^+2^, 0.1 mM dithiothreitol, and 0.17 µM alpha-[^32^P]-dCTP was incubated at 25°C for 20 min. The reaction was stopped by trichloroacetic acid (TCA) precipitation in the presence of 1 µg of yeast tRNA as carriers. The labeled TP was eluted from the gel by cracking and soaking three times in 200 µl of 50 mM ammonium bicarbonate, 5% ß-mercaptoethanol and 0.1% SDS, and precipitated with TCA.

### Phosphoamino Acids Analysis

The procedures of Pargellis *et a*l. [26] and Garcia *et al*. [27] were followed with minor modifications. Purified alpha-[^32^P]-dCMP-labeled Tpg^Sli^ protein was treated in 50 µl of 5.7 N HCl at 110°C for 1–2 h. The hydrolytes were dried and resuspended in 10 µl of water. A sample containing about 50 cpm was mixed with non-radioactive standards (1 µg each phosposerine, phosphothreonine, and phosphotyrosine; Sigma), and spotted on a cellulose thin layer plate (Merck) and subjected to two-dimensional electrophoresis in the Hunter Thin Layer Electrophoresis System (C.B.S. Scientific Company). The first dimension was carried out at pH 1.9 at 1.5 kV for 20 m and the second dimension in pH 3.5 at 1.3 kV for 16 m. The internal standards (marked with dotted circles) were visualized by spraying with 0.25% ninhydrin in acetone, and the radioactivity was imaged by autoradiography.

### Fragmentation of the TP-dCMP Adduct

Alpha-[^32^P]-dCMP-labeled Tpg^Sli^ was isolated from a polyacrylamide gel by elution and precipitation with TCA, and cleaved with CNBr (Sigma) according to the published procedure [28] with minor modifications. The reaction products were separated on a 16% polyacrylamide gel formulated for the analysis of small peptides [29], followed by autoradiography without drying.

For proteolytic digestion with endoproteinase LysC (Roche), alpha-[^32^P]-dCMP-labeled Tpg^Sli^ was isolated using protein A-Sepharose beads, precipitated with TCA, and cleaved with LysC at 25°C for 20 h in a 50-µl solution containing [^32^P]-labeled TP-dCMP, 1 µg enzyme, 25 mM Tris-HCl (pH 8.8), 1 mM EDTA and 0.1 M or 0.3 M of urea, and stopped by vacuum drying. The products were analysis by SDS-PAGE and autoradiography.

### Mass Spectrometric Analysis

Native dCMP-TP complexes for the purpose of mass spectrometric analysis were gained from linear plasmid pLUS980L harbored in *Streptomyces* through four steps of purification as described [19], whereas TpgC was just separated by 12% SDSPAGE because it locates alone against a clear background. Mass spectrometric analysis was operated at either Genomics Center, National Yang Ming University (ESI and MALDI) or Molecular Medicine Research Center, Chang-Gung University (MALDI).

### Construction of Mutated tpg Genes

Two-step PCR was employed to create mutations in *tpg*. The altered sequences were confirmed by sequencing before being used.

### Construction of Linear Plasmids and Test for Linearity of the DNA

Linear plasmids were constructed following the general procedure of Qin *et al*. [30]. The linearity of the plasmid DNA in the transformants was confirmed by restriction digestion and Southern hybridization. To facilitate manipulation of the *tpg* sequence, an *Nde*I site was added immediately upstream of and including the initiation codon of *tpg* on pLUS980. The resulting plasmid was designated pLUS980(Nd). Linear plasmids were generated from these plasmids and their derivative using the previously described procedure [30], [31], *i.e.,* linearization of the plasmid DNA by *Ase*I digestion in the *E. coli* vector sequence followed by transformation of *Streptomyces*.

Various *tpg* homologs, including the putative pseudogenes, were generated by PCR and used to substitute *tpg* ^Sli^ on pLUS980 or pLUS980(Nd) through a series of restriction, subcloning, and ligation manipulations such that *tpg* in the end products was precisely replaced by these sequences without any alteration of the upstream *tap* sequence.

To test the linearity of plasmid DNA, genomic DNA was isolated from *Streptomyces* host (mostly MR04) harboring pLUS980, pLUS980(Nd), or their derivatives, digested with *Sac*I or *Spe*I (which cuts uniquely in these plasmids), electrophoresed in agarose gel, with or without further Southern hybridization. The DNA isolation step involved the use of pronase E, which removed the TP caps on any linear plasmid DNA, allowing it to enter the gel. The linearity is demonstrated by the presence of two restriction fragments of the expected sizes. Circular plasmids, resulting from either incomplete linearization (by *Ase*I digestion) before transformation or spontaneous circularization after transformation, would produce only a restrictions fragment.

### Prediction of Helix-turn-helix Domains and Secondary Structures of Proteins

Helix-turn-helix domain prediction was performed at the Network Protein Sequence Analysis server (http://npsa-pbil.ibcp.fr/cgi-bin/npsa_automat.pl?page=/NPSA/npsa_hth.html) using the method of Dodd and Egan [32]. The GOR method of Garnier *et al*. [33] for protein secondary structure prediction was used as implemented on NPS@ Server (http://npsa-pbil.ibcp.fr/cgi-bin/npsa_automat.pl?page=/NPSA/npsa_server.html).

### Prediction of Hydropathy of Proteins

Protein hydropathy plots were conducted using the Kyte-Doolittle algorithm [34] implemented at the University of Virginia FASTA Server (http://fasta.bioch.virginia.edu/fasta_www2/fasta_www.cgi?rm=misc1).

## Results

### Localization of the Deoxynucleotidylation Site in a C-terminal Fragment of Tpg^Sli^

*In vitro* deoxynucleotidylation of TPs typically selects specifically the nucleotide corresponding to the first nucleotide at the 5′ end of the replicons, such as dCMP for adenovirus-2 [35], dAMP for phage ø29 [21], dCMP for linear plasmid pAL1of *Arthrobacter nitroguajacolicus* [36], dGMP for linear plasmid SCP1 of *Streptomyces* (Tsai, H.-H, unpublished data), and dCMP for the *S. lividans* chromosome [19]. In the case of ø29, the *in vitro* deoxynucleotidylation site was shown to be the attaching site for the telomere DNA *in vivo* [21]. In the case of *S. lividans* chromosome, dCMP was shown to attach to a Thr residue of its TP (designated Tpg^Sli^) in *in vitro* deoxynucleotidylation [19]. Therefore, it was likely that the Thr residue of Tpg^Sli^ deoxynucleotidylated *in vitro* was also deoxynucleotidylated *in vivo*. There are 11 Thr residues on Tpg^Sli^. We set out to determine the deoxynucleotidylated Thr.

**Figure 1:**
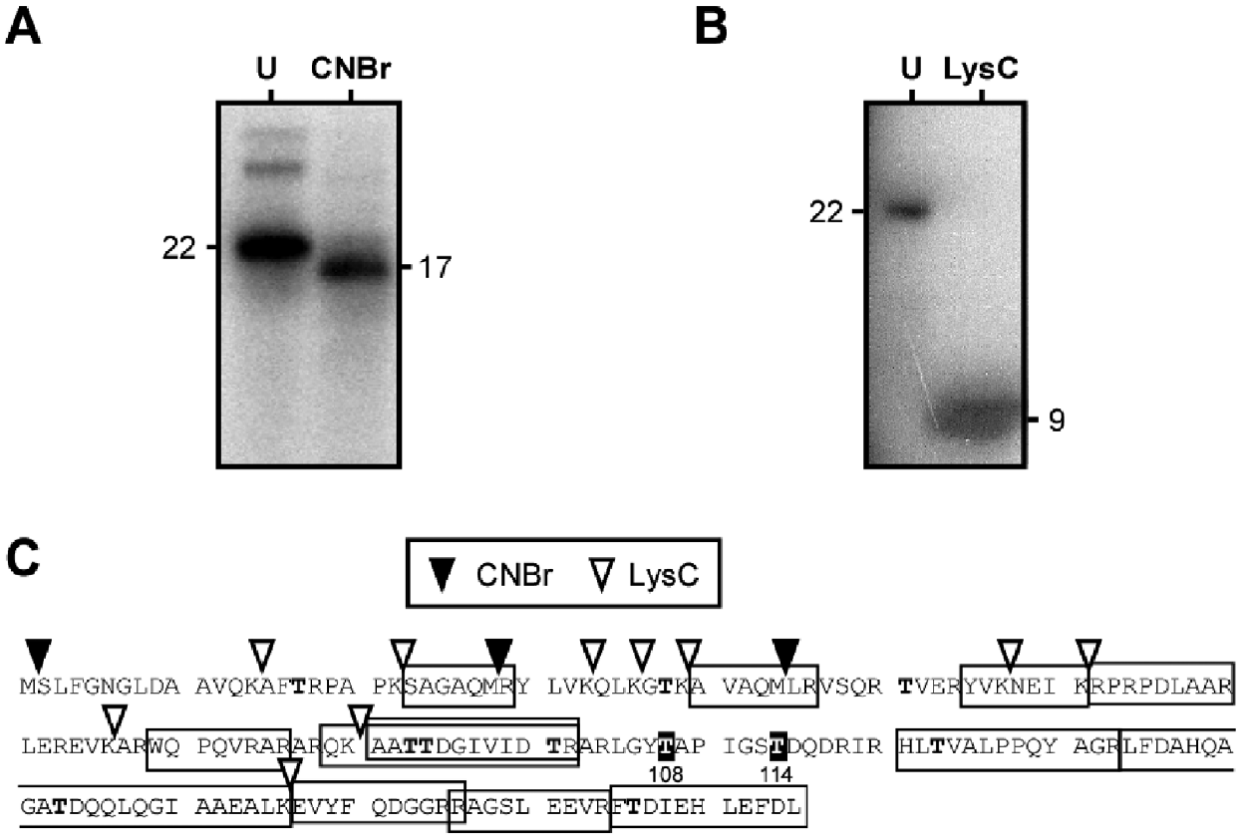
Chemical and enzymatic cleavage of Tpg^Sli^-dCMP. (**A**).Alpha-[^32^P]-dCMP-labeled Tpg^Sli^ was isolated (‘U’), cleaved with CNBr (‘CNBr’), electrophoresed on a 16% polyacrylamide gel in SDS, and the radioactivity detected by autoradiography. (**B**) Alpha-[^32^P]-dCMP-labeled Tpg^Sli^ was isolated (‘U’), and cleaved with LysC (‘LysC’). The digestion products were separated in polyacrylamide gel in SDS and radioactivity was detected by autoradiography. The estimated sizes (in kD) of the radioactively labeled polypeptides are indicated. (**C**) Cleavage map of Tpg^Sli^ protein. The trypsin fragments identified by mass spectrometry [7] are boxed. The cleavage sits of CNBr (filled arrowhead) and LysC (open arrowhead) are marked. The putative deoxynucleotidylated T_108_ and T_114_, suggested by these digestion analyses, are shaded in black. The other Thr residues are in bold face.

The [^32^P]-labeled Tpg^Sli^ protein generated in *in vitro* deoxynucleotidylation was fragmented by CNBr and LysC endoproteinase digestion. Of the three polypeptides produced by CNBr digestion, S_2_∼M_28_ (2.7 kD), R_29_∼M_44_ (1.8 kD), and L_45_∼L_185_ (16.4 kD), the isotope was present in L_45_∼L_185_ (Fig. 1A). In the LysC digest, the isotope was present in the largest polypeptide fragment, which exhibited an apparent molecular weight of about 9 kD (Fig. 1B). The largest LysC polypeptide fragment, A_91_∼K_156_, of Tpg^Sli^, was expected to have a molecular weight of 7.0 kD. We interpreted the increased apparent molecular weight to result from the attachment of the dCMP residue(s). A_91_∼K_156_ was a subset of L_45_∼L_185_ that contained seven Thr residues. These results suggested that the deoxynucleotidylated Thr residue lay in the C-terminal region of Tpg^Sli^.

To narrow down the location of the deoxynucleotidylated Thr in the C-terminal region, we employed mass spectrometry on trypsin-digested Tpg^Sli^ to identify the trypsin fragment that contained the deoxynucleotidylated Thr by its altered molecular weight. Tpg^Sli^–capped DNA was isolated by guanidine HCl-CsCl density gradient centrifugation followed by glass bead binding, and the DNA was trimmed by benzonase, an endonuclease that hydrolyzes DNA into 2- to 3-bp fragments (according to the manufacturer’s specification). The resulting Tpg^Sli^ was isolated by SDS-PAGE, digested with trypsin, and subjected to mass spectrometric analysis by ESI or MALDI ionization. It was anticipated that, if a fragment contained the deoxynucleotidylated Thr, the fragment with the native molecular weight would be absent from the sample (Fig. 1C).

In the C-terminal region, the only expected trypsin fragment that was not detected by either ESI or MALDI was the L_105_∼R_118_ fragment. A fragment with a mass corresponding to that of (dCMP)_2_-L_105_∼R_118_ was detected in the MALDI spectrum. In contrast, trypsin digestion of Tpg^Sli^ protein produced in *E. coli* (presumably without deoxynucleotidylation) gave rise to the expected L_105_∼R_118_ trypsin fragment in both ESI and MALDI spectra. These results indicated that the L_105_∼R_118_ fragment contained the deoxynucleotidylation. The L_105_∼R_118_ fragment lies in the A_91_∼K_156_ LysC fragment and contains two Thr residues, T108 and T114.

### Identification of the Deoxynucleotidylation Site at T114

**Figure 2:**
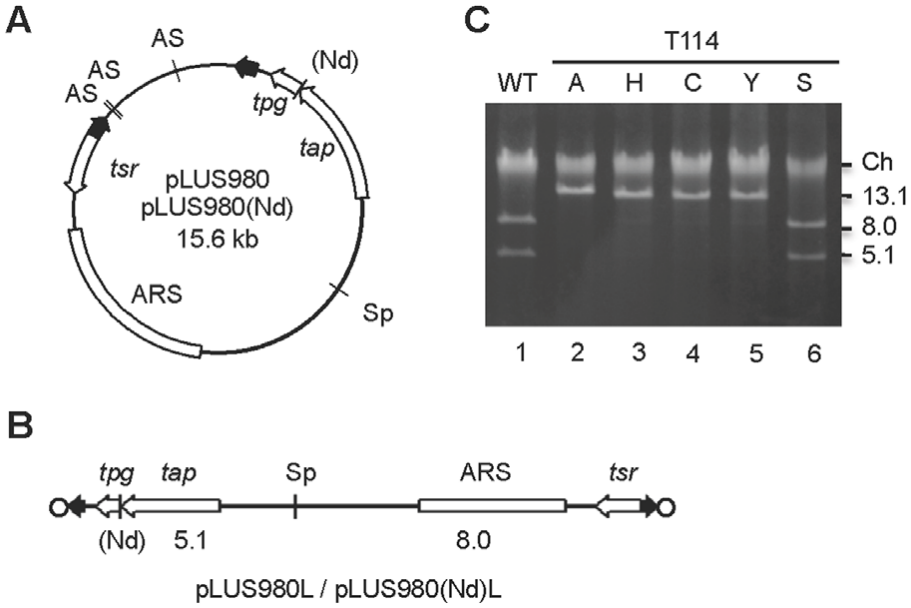
Plasmids pLUS980 and pLUS980(Nd) and their respective linear derivatives pLUS980L, pLUS980(Nd)L. (**A**) Physical maps of pLUS980 and pLUS980(Nd). The open arrows depict the *Streptomyces* genes, and the filled arrows, the 365-bp telomere DNA of the *S. lividans* chromosome. Genetic elements on the *E. coli* vector (short arc between the two telomere sequences) are omitted for clarity. ‘As’ and ‘Sp’, *Ase*I and *Spe*I sites, respectively. An additional *Nde*I site (‘Nd’) is present at the beginning of *tpg* ^Sli^ in pLUS980(Nd), but not in pLUS980. ARS, autonomous replication sequence from pSLA2 [30]. (**B**) Physical map of pLUS980L and pLUS980L(Nd). The Tpg^Sli^ that caps the linear plasmids is depicted as open circles. Other symbols are as in **A**. The size of the *Spe*I fragments is given in kb. (**C**) The genomic DNA from transformants of *Ase*I-linearized pLUS980(Nd) derivatives was digested by *Spe*I, and separated by electrophoresis. Lane 1, wild type *tpg* ^Sli^. Lanes 2–5, *tpg* ^Sli^ containing a substitution of T114 by Ala, His, Cys, Tyr, or Ser, respectively. The sizes (in kb) of the restriction fragments are indicated. ‘Ch’, chromosomal fragments.

Site-directed mutagenesis was subsequently employed to replace T108 and T114 with different residues in *tpg* ^Sli^ to test the functionality of the resulting mutant Tpg^Sli^. To do this, the mutant *tpg* gene was placed on pLUS980 or its derivative pLUS980(Nd) (Fig. 2A). These two plasmids contained a linear plasmid sequence consisting of a pair of telomeres of the *S. lividans* chromosome flanking a thiostrepton resistance gene (*tsr*), an autonomously replicating sequence (ARS) of linear plasmid pSLA2, and the *tap*-*tpg* operon of the *S. lividans* chromosome. These two plasmids, upon linearization (by *Ase*I digestion in the *E. coli* sequence) and transformation [30], [31], generated linear plasmids with Tpg-capped telomeres, designated pLUS980L and pLUS980(Nd)L, respectively (Fig. 2B).

*S. lividans* MR04, in which the *tap^Sli^*-*tpg^Sli^* operon was deleted along with large stretches of terminal DNA from the chromosome [23], was chosen as the host. If the *tpg* variant on pLUS980 and pLUS980(Nd) were defective, transformation of MR04 using the *Ase*I-linearized plasmid DNA would produce either no transformants, or a few transformants harboring only circular plasmids. The latter would result from the plasmids that had escaped *Ase*I digestion or linear fragments that had circularized in the transformants [37]. The topology of the plasmids in the transformants was determined by restriction digestion. For example, *Spe*I, which cuts singly in pLUS980 and pLUS980(Nd) DNA, would produce two fragments of 5.1 and 8.0 kb from the linear plasmid DNA, but would produce a single larger fragment from the circular plasmid DNA (Fig. 2C).

Firstly, T108 and T114 were individually mutated to Ser in Tpg^Sli^. Interestingly, both the resulting plasmids, pLUS980-T108S (Fig. S1 in Supporting Information) and pLUS980-T114S (Fig. 2C, lane 6), could replicate in linear form in MR04, indicating that neither of the mutations inactivated Tpg^Sli^. Similarly, the T108S and T114S mutations were individually created in *tpg* ^SLP2.19^, and again these mutations did not inactivate Tpg^SLP2.19^ (data not shown). These results suggested that either none of these Thr residues was the deoxynucleotidylation site, or the deoxynucleotidylated Thr could be substituted by a Ser without losing its function. Substitutions of four other Thr residues at 101, 123, 143 and 176 positions in the C-terminal region also did not destroy the ability of Tpg to support replication of the linear plasmid (Fig. S2 in Supporting Information).

Next, T108 and T114 of Tpg^Sli^ were substituted by three other residues – Ala, Cys, and Tyr. None of these substitutions at T108 inactivated Tpg^Sli^. In contrast, the substitutions at T114 gave rise to transformants at very low frequencies (about three orders of magnitude lower), which harbored only circular plasmids (Fig. 2C, lanes 2, 4, 5). Substitution of T114 by a His, which might provide an amino group to form a covalent bond with the nucleotide [38], also failed to support replication of the linear plasmid (Fig. 2C, lane 3). These results eliminated the role of T108 as the site of deoxynucleotidylation, leaving T114 as the final candidate.

**Figure 3:**
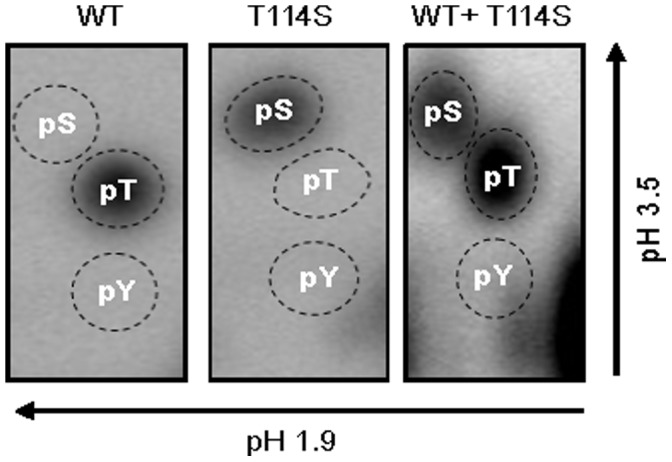
Determination of the deoxynucleotidylated amino acids. The alpha-[^32^P]-dCMP-labeled Tpg protein obtained from *in vitro* deoxynucleotidylation subjected to acid hydrolysis, followed by two-dimensional electrophoresis together with internal standards of non-radioactive phosphoserine, phosphothreonine, and phosphotyrosine. *Left panel*, hydrolysate prepared from deoxynucleotidylated wild-type Tpg^Sli^. *Middle panel*, hydrolysate prepared from deoxynucleotidylated Tpg^Sli^ containing T114S substitution. *Right panel*, hydrolysate prepared from deoxynucleotidylated mixture of equal weights of wild-type Tpg^Sli^ and Tpg^Sli^ containing T114S substitution.

If T114 was the deoxynucleotidylation site *in vivo*, the Ser residue that substituted it in the T114S mutants would be expected to be deoxynucleotidylated, and this was tested in an *in vitro* deoxynucleotidylation assay using His-tagged Tpg^Sli^ and Tpg^Sli^-T114S proteins produced in *E. coli*. In the *in vitro* deoxynucleotidylation reaction, approximately equal weights of Tpg^Sli^ and Tpg^Sli^-T114S were labeled by radioactive dCMP. The alpha-[^32^P]- dCMP labeled Tpg^Sli^ and Tpg^Sli^-T114S were subjected to acid hydrolysis, and chromatography on cellulose thin layer plate [19]. The results showed that the labeled dCMP was attached to a Ser residue on Tpg^Sli^-T114S (Fig. 3 middle panel), while it was attached to a Thr residue of Tpg^Sli^ (Fig. 3. left panel). The efficiency of deoxynucleotidylation for Tpg^Sli^-T114S was approximately 30% of that for Tpg^Sli^ (Fig. 3, right panel). These results supported the idea that T114 was the deoxynucleotidylation site *in vivo* and that it could be functionally substituted by Ser albeit possibly with a reduced efficiency of deoxynucleotidylation.

In the Tpg^Sli^ sequence, T114 is followed closely by two negatively charged Asp residues (D115 and D117 in Tpg^Sli^). In the ø29 TP, the deoxynucleotidylated S232 is also flanked by two negatively charged Asp (D231) and Glu (E233). Introduction of a D115A mutation destroyed the Tpg function, whereas a D117A mutation had no effect on the Tpg function (Fig. S3B, Supporting Information). Interestingly, D115 could be substituted by a Glu (D115E mutation) but not by a Asn (D115N mutation) without destroying the Tpg function (Fig. S3B, Supporting Information), indicating that a negative charge there is important.

The residue upstream of T114 is also a Ser. A S113A mutation had no effect on the Tpg functionality (Fig. S1 in Supporting Information).

### Testing Tpg Variants for Support of Replication of Linear Plasmids

**Figure 4:**
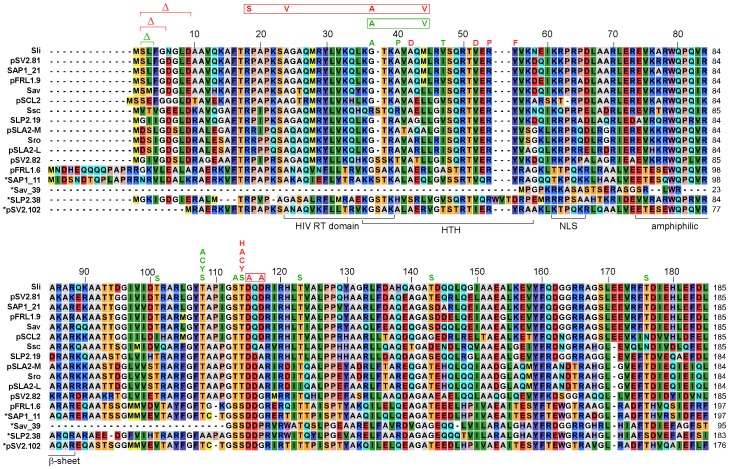
Tpg sequence analysis. The sequences of Tpg homologs encoded by various *Streptomyces* chromosomes and linear plasmids, including translation products of pseudogenes, are aligned. The chromosome-encoded Tpgs are designated by three-letter abbreviations of the species (Sli, *S. lividans*; Sav, *S. avermitilis*; Sro, *S. rochei*; Ssc, *S. scabies*), the plasmid-encoded Tpgs by the plasmid names and the pseudogenes by the designations in the sequence databases or publications. Tpgs encoded by the same replicon are distinguished by their gene designations. Sources of the sequences are: *S. coelicolor* chromosome [7], *S. lividans* chromosome [6], *S. avermitilis* chromosome and SAP1 plasmid [10], pSV2 plasmid (pSV2.82) in *S. violaceoruber* (GenBank accession number NC_004934), pFRL1 plasmid in *Streptomyces* sp. FR1 [45], *S. rochei* chromosome and pSLA2-L and pSLA2-M plasmids [46], SLP2 plasmid in *S. lividans* [7], *S. scabies* chromosome (http://www.sanger.ac.uk/Projects/S_scabies/), pSCL2 plasmid in *S. clavuligerus* (GenBank accession number AY392421). The amino acid numbering is that of Tpg^Sli^. The lengths (in amino acid residues) of the Tpgs are indicated at the right. Conceptually translated products of pseudogenes proven in this study (SAP1_11, SLP2.38, and pSV2.102) and the apparent pseudogene, Sav_39 (with deletions of about 90 amino acids), are marked by asterisks. The structural and functional domains previously identified are indicated at the bottom: HIV reverse transcriptase (RT) domain; helix-turn-helix (HTH) domain; nuclear localization signal (NLS); and amphiphilic beta-sheet. Substitution mutations investigated in this study are placed above the sequence. Deletions are indicated by ‘Δ’. Multiple substitutions are boxed together. Mutations that inactivated Tpg are shown in red; those that did not, in green.

Examination of the aa sequences of 17 Tpg homologs (including the conceptual translation products of the putative pseudogenes; Fig. 4) revealed that 12 contained T114 and five contained S114. Of the five S114-containing *tpg* homologs, *tpg* ^Sav_39^, which encodes a product of only about one half length (95 aa), is most likely a pseudogene. The other four S114-containing homologs, *tpg* ^SLP2.38^, *tpg* ^pFRL1.6^, *tpg* ^SAP1_11^, and *tpg* ^pSV2.102^, along with the T114-containing *tpg* ^SLP2.19^ were tested for their ability to support the replication of linear plasmids. All these *tpg* homologs are present on a linear plasmid unaccompanied by a *tap* homolog. Of these, *tpg* ^SLP2.19^ and *tpg* ^SLP2.38^ are on the same (SLP2) plasmid [9], with *tpg* ^SLP2.19^ lying in the left arm and *tpg* ^SLP2.38^ lying in the 15.4-kb right arm sequence, which is shared by the ends of the *S. lividans* chromosome [39].

These *tpg* homologs were placed on pLUS980 and pLUS980(Nd), and tested for their ability to support linear replication in MR04. Of the five putative pseudogenes tested, *tpg* ^SLP2.38^, *tpg* ^SAP1_11^, and *tpg* ^pSV2.102^ could not support replication of linear plasmids in MR04 (Fig. S4 in Supporting Information), indicating that they were indeed pseudogenes. *tpg* ^SLP2.19^ and *tpg* ^pFRL1.6^ could support replication of linear plasmids in MR04 (Fig. S4 in Supporting Information). It was probably not surprising that these two genes are functional, because the encoded Tpg homologs are relatively conserved and the codon usage is typical for *Streptomyces* (high G/C at the third positions of the codons). However, Tpg^pFRL1.6^ contains a 13-amino acid extension at the N-terminus. Interestingly, the pseudogene product Tpg^SAP1_1^ is highly similar to Tpg^pFRL1.6^ in sequence including the 13-amino acid extension at the N-terminus. The defect of this product was likely to be due to one or more amino acid substitutions elsewhere.

*tpg* ^SLP2.19^, when placed downstream of *tap* on pLUS980L, was presumably transcribed from the same promoter as *tap*. To test whether the lone *tpg* ^SLP2.19^ on SLP2 was expressed, and, if so, whether it alone was sufficient for supporting the replication of SLP2, attempts were made to use SLP2tsr, an SLP2 derivative with *tsr* inserted in Tn*4811* [39], to transform MR04 (Δ*tap-tpg*) and ZX7 (parent of MR04, *tap-tpg* ^+^). Thio^r^ transformants were readily obtained in ZX7, but not in MR04. This indicated that either *tpg* ^SLP2.19^ was not expressed on SLP2, or the participation of *tap* was required as shown in the replication of the *Streptomyces* chromosomes [8]. To check this, *tap* ^Sli^ was inserted into the øC31 *att* site on the MR04 chromosome using the integrative plasmid pSET152 [40]. The resulting strain, CK03, could then be transformed successfully with SLP2tsr. This result indicated that *tpg* ^SLP2.19^ on SLP2 was functional, and the presence of *tap* elsewhere was required for the replication of SLP2.

### Analysis of the N-terminal Region of Tpg

Compared to most other Tpgs, the pseudogene product Tpg^pSV2.102^ lacks eight amino acid residues at the N-terminus (Fig. 4). To examine the importance of these eight amino acids, three mutations in Tpg^Sli^ were created with a deletion of eight (residue 2 to 9), four (2 to 5), and two (2 to 3) amino acids from the N-terminus, and the effects of these deletions were tested. The results showed that, the deletions of eight and four amino acids inactivated Tpg^Sli^, but that of two amino acids did not (Fig. S5, Supporting Information).

In addition to the N-terminal truncation, the 47^th^ residue (based on Tpg^Sli^) of Tpg^pSV2.102^, located in the ‘turn’ of the predicted HTH motif of Tpg, is a Thr, while Ala or Ile are present at this position in the other Tpgs (Fig. 4). To test the possible effect of the Thr substitution, a V47T mutation was introduced into Tpg^Sli^. The resulting Tpg^Sli^ was functional, indicating that the V47T substitution had not effect on the functionality of Tpg^Sli^.

Tpgs possess a putative helix-turn-helix (HTH) motif despite the fact that the *in vitro* DNA-binding specificity appears to be low [6](Yi-Hong Chen, unpublished results). The atypical TP, Tpc, also contains a HTH domain with a different sequence [18]. To examine the importance of the predicted HTH domain in Tpg sequences (Fig. 4), several amino acids in the helix domains of Tpg^Sli^ were substituted by different residues to lower their potential to form HTH (Table 1). In the first helix of the HTH motif, the G37A and A40P mutations (which reduced the probability of HTH formation to 25%) did not cause a defective Tpg. Quadruple mutation, R18S-A26V-G37A-M44V, generated by error-prone PCR, inactivated Tpg^Sli^ (data not shown). Of these four mutations, G37A and M44V were in the HTH motif. Tpg^Sli^ containing the G37A-M44V double mutation had a 71% probability of HTH formation, and exhibited no effect. Therefore, the defect caused by the quadruple mutations must be attributed to the other two upstream mutations. Moreover, an A42D mutation (insignificant probability of HTH formation) inactivated Tpg^Sli^ (Fig. S6, Supporting Information). Perhaps the collapse of the first helix might not be critical, but the defect was caused by repulsion of DNA by the negative charge of the Asp residue. These results are summarized in Table 1 and Fig. 4.

**Table 1:** Effects of mutations in the putative HTH region of Tpg^Sli^ on the probability of HTH formation and ability to support replication of linear plasmids.

Mutation	Sequence^1^	% Probability of HTH	Plasmid linearity^2^
Wild-type	KGTKAVAQMLRVSQRTVERYVK	100	+
G37A	KATKAVAQMLRVSQRTVERYVK	25	+
G37A M44V	KATKAVAQVLRVSQRTVERYVK	71	+
A40P	KGTKPVAQMLRVSQRTVERYVK	25	+
A42D	KGTKAVDQMLRVSQRTVERYVK	Insignificant	−
V47T	KGTKAVAQMLRTSQRTVERYVK	90	+
V52D	KGTKAVAQMLRVSQRTDERYVK	71	−
R54P	KGTKAVAQMLRVSQRTVEPYVK	71	−
Y55F	KGTKAVAQMLRVSQRTVERFVK	90	−

1 The substituted amino acid residues are underlined.

2 Support of linear plasmid replication: ‘+’, yes; ‘−’, no.

The second helix of the HTH motif is more hydrophilic. The R54P and Y55F mutations (71% and 90% probability of HTH formation, respectively) resulted in a defective Tpg^Sli^ (Fig. S6, Supporting Information). Possibly the positively charge R54 was involved in interaction with a phosphate group on the DNA. A V52D mutation in this region that introduced a negatively charged amino acid and reduced the predicted probability of HTH formation to 71% also resulted in a defective Tpg^Sli^ (Fig. S6, Supporting Information).

These results indicated the importance of the HTH motif of Tpg^Sli^ in end patching. This is in contrast to the NLS motif that immediately follows the HTH motif, which may be mutated without affecting replication of the linear plasmids [15],

## Discussion

In this study, we have identified T114 to be the site of deoxynucleotidylation of Tpg^Sli^. A substitution of T114 with Tyr, Cys, or His inactivated Tpg^Sli^. A substitution with Ser did not, which is not surprising, because the functional lone Tpg^pFRL1.6^ also contains a Ser at this position (Fig. 4). For ø29 TP, a substitution of the deoxynucleotidylated residue S232 with Thr inactivates the TP [41], but a substitution with Cys produces a TP with a reduced (7%) efficiency of deoxynucleotidylation [42].

It is noteworthy that the amino acid residue immediate upstream of T114 is also a Ser. When T114 became defective in deoxynucleotidylation through a His/Ala/Cys/Tyr substitution, S113 could not serve as the deoxynucleotidylation site as S114 could. Thus, it appeared that, while Ser might serve as a deoxynucleotidylation, the deoxynucleotidylation is specific at position 114.

**Figure 5:**
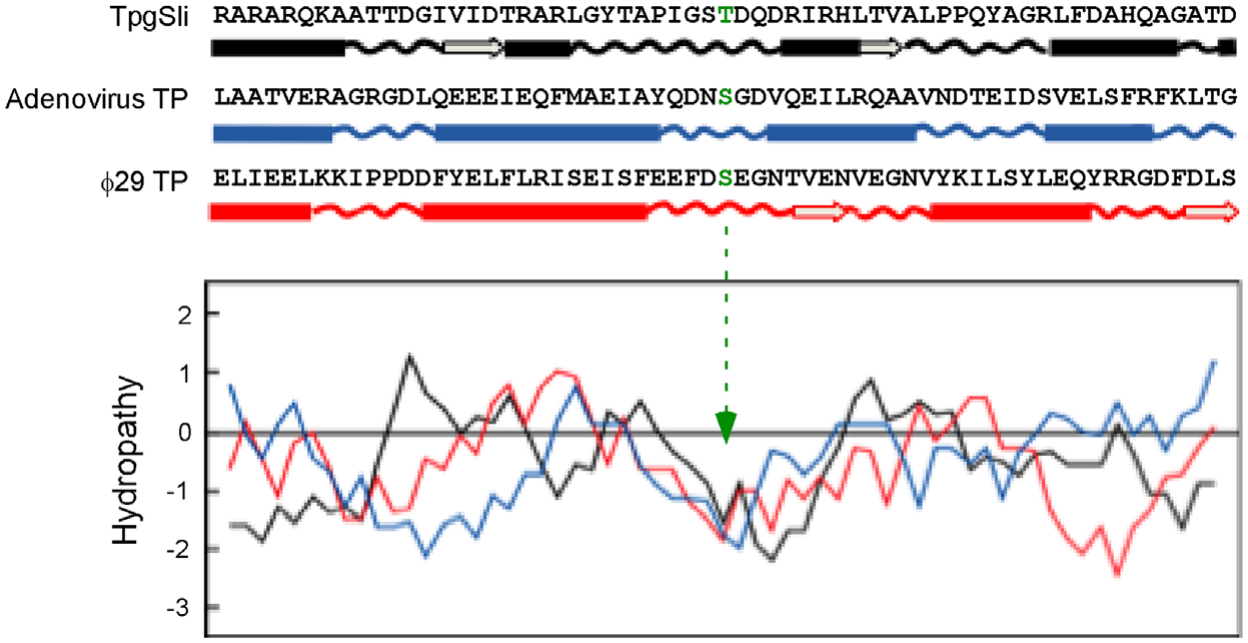
Hydropathy and secondary structural analyses at the deoxynucleotidylation sites. The deoxynucleotidylation sites and neighboring 60 amino acid residues in Tpg^Sli^, adenovirus TP, and ø29 TP were analyzed. (*Top*) The predicted secondary structures. Bars, alpha-helixes; wavy lines, coils; open arrows, beta-strands. The deoxynucleotidylated amino acids are shown in green. (*Bottom*) Hydropathy plots. Window size: 9. The positions of the deoxynucleotidylation sites in the center are marked by the dashed green arrow. Black, Tpg^Sli^; blue, adenovirus TP; red, ø29 TP.

The different effects of T114Y and T114S substitutions are interesting. While both Tyr and Ser possess a priming hydroxyl group for DNA synthesis, the inability of Tyr to serve the priming function may be due to its bulkier and relatively more hydrophobic side chain. Secondary structure predication by the methods of Garnier, *et al*. [33] places T114 in a coiled domain flanked by two short helix segments in Tpg^Sli^ (Fig. 5 top). This is similar to the situations of the TPs of ø29 and adenoviruses. That the deoxynucleotidylated Ser residues in these two viral TPs lie in a predicted coil segment flanked by two short helixes has been previously noticed [21]. In the case of the TPs of ø29, this prediction was confirmed in the TP-DNA polymerase c-crystal structure [44].

Hydropathy prediction showed that T114 lay in a highly hydrophilic region, presumably close to the surface of the protein (Fig. 5 bottom). The deoxynucleotidylated S232 of ø29 [45] and S557 of adenovirus [20] also lie in highly hydrophilic regions (Fig. 5 bottom). In contrast to these, the deoxynucleotidylated Y190 of PRD1 TP lies in a ß-structure enclosed in a hydrophobic area [45].

Despite these similarities in geometric properties, there is no similarity in sequence and size among the TPs in the different systems. The patching TP-primed DNA synthesis in *Streptomyces* also differs from the replicative TP-primed DNA synthesis (ø29, adenovirus, and PRD1) in that the former requires a proper DNA template for *in vitro* deoxynucleotidylation [19], whereas the latter does not. Moreover, the telomere sequences in the former consist of extensive palindromic sequences with potentials of forming complex secondary structures, which is not found in the telomeres of the latter. It follows that the telomere associating protein, Tap, that recognizes the secondary structure formed by the 3′ overhang at the telomeres is also absent from the replicative TP-primed synthesis systems. All these considerations, plus the use of different DNA polymerases by these systems [43], lead to a conclusion that these different TP-primed DNA synthesis systems have evolved independently, recruiting different proteins with a suitable priming function in the process. Even among *Streptomyces*, there are atypical, heterologous TPs capping linear plasmids and linear chromosomes, one of which (Tap of SCP1) has been characterized [18].

In addition to the variations in TP families in *Streptomyces*, the frequent occurrence of *tpg* pseudogenes is also remarkable for an essential housekeeping gene. In this study, we have demonstrated three defective *tpg* pseudogenes out of five candidates. It is unlikely that the pseudogenes have arisen by gene duplication, and the absence of an accompanying *tap* gene argues against it. More likely, the lone *tpg* homologs were acquired through horizontal transfer from a plasmid. The findings of the *tpg* pseudogenes in the terminal regions of the linear replicons of *Streptomyces*, where exchanges with other linear replicons are frequent, also support this notion. The abundance of *tpg* pseudogenes suggests that such horizontal events are relatively frequent.

SLP2 is interesting in that it contains two lone *tpg* homologs, the functional *tpg^SLP2.19^* and the pseudogene *tpg^SLP2.38^*. Although *tpg^SLP2.19^* is functional, a *tap* gene present elsewhere is nonetheless required for the replication of SLP2. This finding answers the previous observations that SLP2 could not replicate in certain *S. lividans* mutants, whose chromosomes had suffered circularization and deletions (in the terminal regions where the *tap-tpg* operon resides) [44]. Apparently, *tap* was the chromosomal gene in the deletion that was required for replication of SLP2. The requirement of *tap* for replication of SLP2 is the same for that of the linear chromosomes in *Streptomyces* [8]. The evolutionary significance of the emergence of lone functional *tpg* is obscure.

## Supporting Information

Figure S1 **Topology of pLUS980(Nd) derivatives containing substitutions at T108 or T114 of** ***tpg*** **.** (*A*) Physical maps of plasmids pLUS980 and pLUS980(Nd). (*B*) Genomic DNA containing a pLUS980 derivative (except lanes *2* and *3*) was isolated from MRO4, digested with *Spe*I (Sp), and subjected to agarose gel electrophoresis. The four substitutions at T108 and at T114 are indicated by the substituting amino acids. Lane *M*, I kb DNA ladders as size markers. Lanes *1–5*, plasmid DNA (purified or in total genomic DNA) of known topology and size serving as controls and markers: *1*, *Spe*I-digested genomic DNA from MR04 containing pLUS980L (fragment size: 8.0 and 5.1 kb); *2, Ase*I-digested pLUS980 (largest fragment 14.3 kb); *3*, *Spe*I*-*digested pLUS980 (15.6 kb); lane *4*: *Spe*I-digested genomic DNA from MR04 containing *Spe*I-digested pLUS980(Nd)L (8.0 and 5.1 kb); *5*, *Spe*I-digested genomic DNA from MR04 containing pLUS980(Nd)L with a S113A substitution in *tpg* (8.0 and 5.1 kb). The sizes (in kb) of the *Spe*I fragments are indicated. The sizes of the circularized plasmid DNA derived from the four linearized pLUS980(Nd) derivatives with a substitution at T114 varied from transformants to transformants, depending on the end joining sites. Shown here are representative cases.
(TIFF)

Figure S2 **Topology of pLUS980(Nd) derivatives containing a T101S, T143S, T176S, or T123S substitution in** ***tpg*** **.** (*A*) Physical maps of plasmids pLUS980 and pLUS980(Nd). (*B*) Isolated genomic DNA was digested with *Sac*I (Sa) except for that in lane *2*, which was not enzyme digested. The sizes (in kb) of pLUS980(Nd)L (T143S) and the *Sac*I fragments are indicated.
(TIFF)

Figure S3 **Topology of the pLUS980(Nd) derivatives containing D115A and D117A mutations.** (*A*) Physical maps of plasmids pLUS980 and pLUS980(Nd). (*B*) Genomic DNA from transformants of pLUS980(Nd) and its derivatives linearized by *Ase*I digestion. Lane 1, pLUS980(Nd). Lane 2, pLUS980(Nd) containing D115A mutation (no linear plasmid present). Lane 3, pLUS980(Nd) containing D117A mutation. Lane 4, pLUS980(Nd) containing D115E mutation. Lane 5, pLUS980(Nd) containing D115N mutation (no linear plasmid present). The sizes of the circularized plasmid DNA derived from the transformants varied from transformants to transformants, depending on the end joining sites. M, 1-kb DNA markers.
(TIFF)

Figure S4 **Topology of pLUS980 derivatives containing** ***tpg*** **homologs from linear plasmids.** (*A*) Physical maps of plasmids pLUS980 and pLUS980(Nd). (*B*) Genomic DNA isolated from MR04 transformants of the pLUS980 derivatives, and electrophoresed with or without prior restriction digestion with *Sac*I or *Spe*I as indicated. *tpg* ^Sli^ was substituted by the following homologs in the pLUS980 derivatives: *tpg* ^SLP2.19^ **(**pCY72L), *tpg* ^SLP2.38^ **(**pCY73), *tpg^S^* ^AP1-11 (^pCY77N), *tpg* ^pSV2.102^_(_pCY78N)^,^ *tpg^p^* ^FRL1.6^_(_pCY79N). M1, 1-kb maker DNA; M2, 14.2-kb *Ase*I fragment from pLUS980(Nd) DNA. The sizes of the circular plasmid DNA derived from circularization of linearized pCY73, pCY77N, and pCY78N varied from transformants to transformants, depending on the end joining sites. Shown here are representative cases.
(TIFF)

Figure S5 **Topology of pLUS980(Nd) derivatives containing N-terminal deletions in** ***tpg*** **.** (*A*) Physical maps of plasmids pLUS980 and pLUS980(Nd). (*B*) Genomic DNA from three independent transformants of MR04 was digested with *Sac*I (Sa). Lanes 1–3, deletion of 2 aa’s (residues 2–3); lanes 4–6, deletion of 4 aa’s (residues 2–5); lane 7–9, deletion of 8 aa’s (residues 2–9); lane 10, pLUS980(Nd)L. The sizes (in kb) of the *Sac*I fragments are indicated. The sizes of the circularized plasmid DNA derived from the transforming linear plasmid DNA varied from transformants to transformants, depending on the end joining sites. Shown here are representative cases.
(TIFF)

Figure S6 **Topology of pLUS980(Nd) derivatives containing mutations in the HTH domain of** ***tpg*** **.** (*A*) Physical maps of plasmids pLUS980 and pLUS980(Nd). (*B*) Genomic DNA isolated from MR04 transformants containing a pLUS980(Nd) derivative was digested with *Sac*I (Sa) and electrophoresized. The mutations (V47T, V52D) in *tpg* are indicated. pLUS980(Nd) DNA digested by *Ase*I (As) and 1-kb DNA ladder (‘M’) are used as size markers. (*C*) Genomic DNA isolated from MRO4 transformants were digested with *Spe*I (Sp), electrophoresized, and hybridized with pLUS980 DNA as probe. The mutations in *tpg* are indicated. The sizes of the circularized plasmid DNA derived from the transforming linear plasmid DNA varied from transformants to transformants, depending on the end joining sites. Shown here are representative cases.
(TIFF)
