# Supplementary figures and images for: Mutational Analysis of the Terminal Protein Tpg of Streptomyces Chromosomes: Identification of the Deoxynucleotidylation Site
Source: PLoS One. 2013 Feb 14;8(2):e56322. doi: 10.1371/journal.pone.0056322 (PMC3572947; doi:10.1371/journal.pone.0056322)

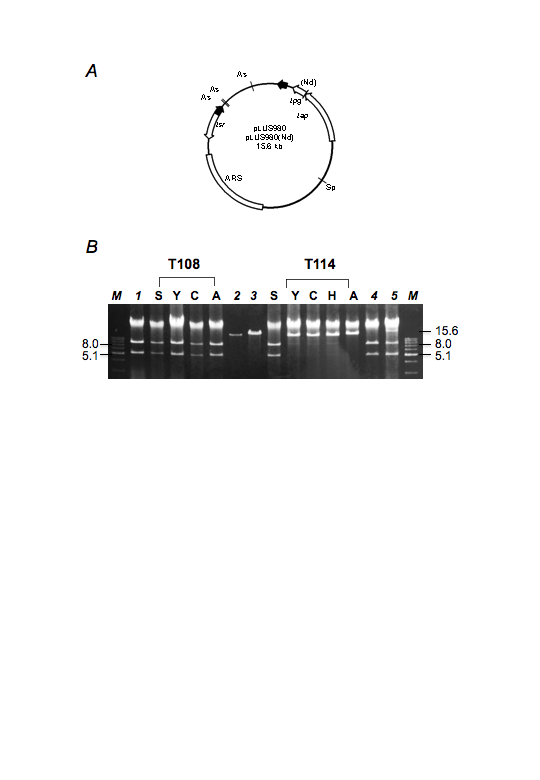

Supplement: Figure S1 — Topology of pLUS980(Nd) derivatives containing substitutions at T108 or T114 of tpg . (A) Physical maps of plasmids pLUS980 and pLUS980(Nd). (B) Genomic DNA containing a pLUS980 derivative (except lanes 2 and 3) was isolated from MRO4, digested with SpeI (Sp), and subjected to agarose gel electrophoresis. The four substitutions at T108 and at T114 are indicated by the substituting amino acids. Lane M, I kb DNA ladders as size markers. Lanes 1–5, plasmid DNA (purified or in total genomic DNA) of known topology and size serving as controls and markers: 1, SpeI-digested genomic DNA from MR04 containing pLUS980L (fragment size: 8.0 and 5.1 kb); 2, AseI-digested pLUS980 (largest fragment 14.3 kb); 3, SpeI-digested pLUS980 (15.6 kb); lane 4: SpeI-digested genomic DNA from MR04 containing SpeI-digested pLUS980(Nd)L (8.0 and 5.1 kb); 5, SpeI-digested genomic DNA from MR04 containing pLUS980(Nd)L with a S113A substitution in tpg (8.0 and 5.1 kb). The sizes (in kb) of the SpeI fragments are indicated. The sizes of the circularized plasmid DNA derived from the four linearized pLUS980(Nd) derivatives with a substitution at T114 varied from transformants to transformants, depending on the end joining sites. Shown here are representative cases. (TIFF) [file pone.0056322.s001.tif]

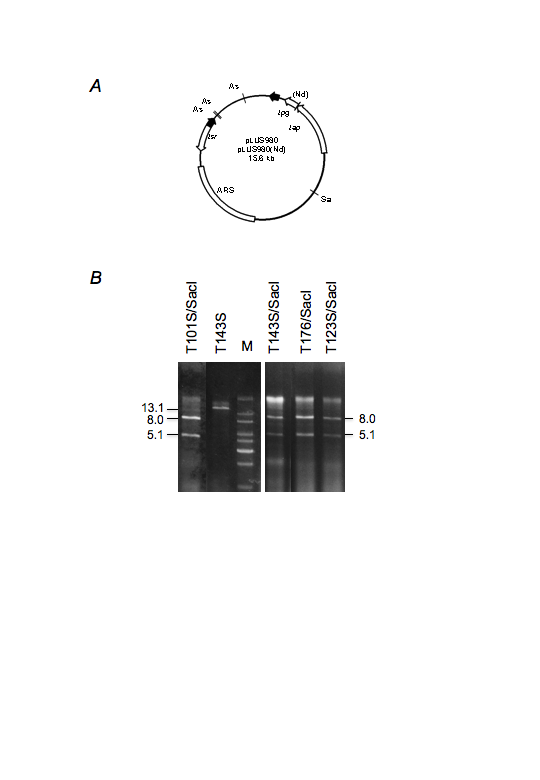

Supplement: Figure S2 — Topology of pLUS980(Nd) derivatives containing a T101S, T143S, T176S, or T123S substitution in tpg . (A) Physical maps of plasmids pLUS980 and pLUS980(Nd). (B) Isolated genomic DNA was digested with SacI (Sa) except for that in lane 2, which was not enzyme digested. The sizes (in kb) of pLUS980(Nd)L (T143S) and the SacI fragments are indicated. (TIFF) [file pone.0056322.s002.tif]

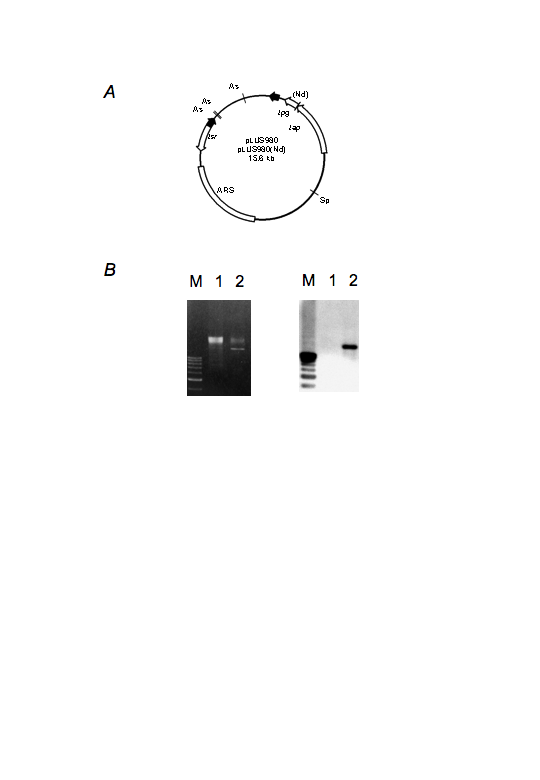

Supplement: Figure S3 — Topology of the pLUS980(Nd) derivatives containing D115A and D117A mutations. (A) Physical maps of plasmids pLUS980 and pLUS980(Nd). (B) Genomic DNA from transformants of pLUS980(Nd) and its derivatives linearized by AseI digestion. Lane 1, pLUS980(Nd). Lane 2, pLUS980(Nd) containing D115A mutation (no linear plasmid present). Lane 3, pLUS980(Nd) containing D117A mutation. Lane 4, pLUS980(Nd) containing D115E mutation. Lane 5, pLUS980(Nd) containing D115N mutation (no linear plasmid present). The sizes of the circularized plasmid DNA derived from the transformants varied from transformants to transformants, depending on the end joining sites. M, 1-kb DNA markers. (TIFF) [file pone.0056322.s003.tif]

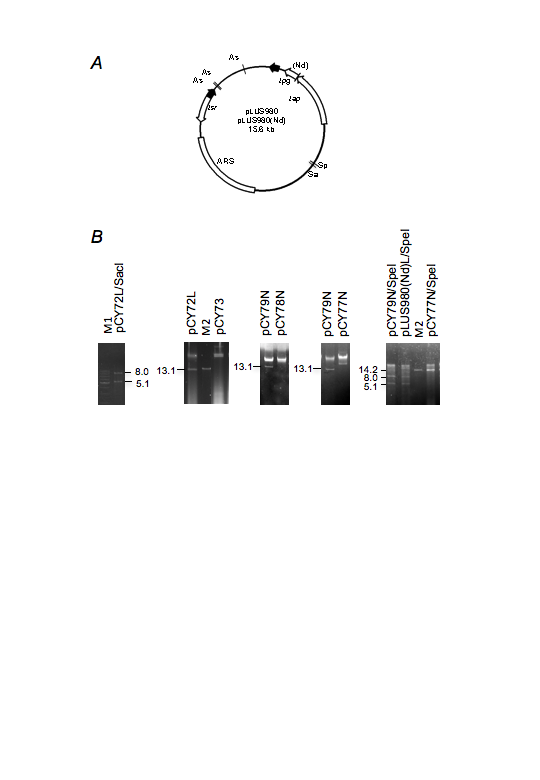

Supplement: Figure S4 — Topology of pLUS980 derivatives containing tpg homologs from linear plasmids. (A) Physical maps of plasmids pLUS980 and pLUS980(Nd). (B) Genomic DNA isolated from MR04 transformants of the pLUS980 derivatives, and electrophoresed with or without prior restriction digestion with SacI or SpeI as indicated. tpg Sli was substituted by the following homologs in the pLUS980 derivatives: tpg SLP2.19 (pCY72L), tpg SLP2.38 (pCY73), tpgS AP1-11 (pCY77N), tpg pSV2.102 (pCY78N), tpgp FRL1.6 (pCY79N). M1, 1-kb maker DNA; M2, 14.2-kb AseI fragment from pLUS980(Nd) DNA. The sizes of the circular plasmid DNA derived from circularization of linearized pCY73, pCY77N, and pCY78N varied from transformants to transformants, depending on the end joining sites. Shown here are representative cases. (TIFF) [file pone.0056322.s004.tif]

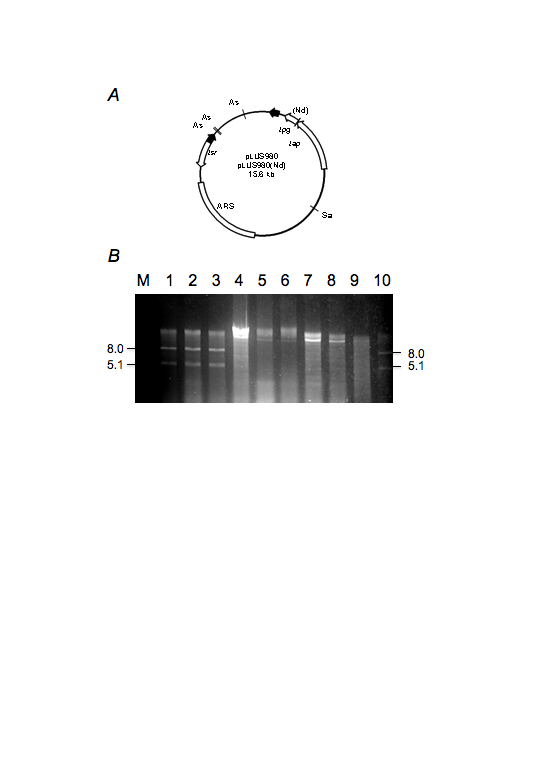

Supplement: Figure S5 — Topology of pLUS980(Nd) derivatives containing N-terminal deletions in tpg . (A) Physical maps of plasmids pLUS980 and pLUS980(Nd). (B) Genomic DNA from three independent transformants of MR04 was digested with SacI (Sa). Lanes 1–3, deletion of 2 aa’s (residues 2–3); lanes 4–6, deletion of 4 aa’s (residues 2–5); lane 7–9, deletion of 8 aa’s (residues 2–9); lane 10, pLUS980(Nd)L. The sizes (in kb) of the SacI fragments are indicated. The sizes of the circularized plasmid DNA derived from the transforming linear plasmid DNA varied from transformants to transformants, depending on the end joining sites. Shown here are representative cases. (TIFF) [file pone.0056322.s005.tif]

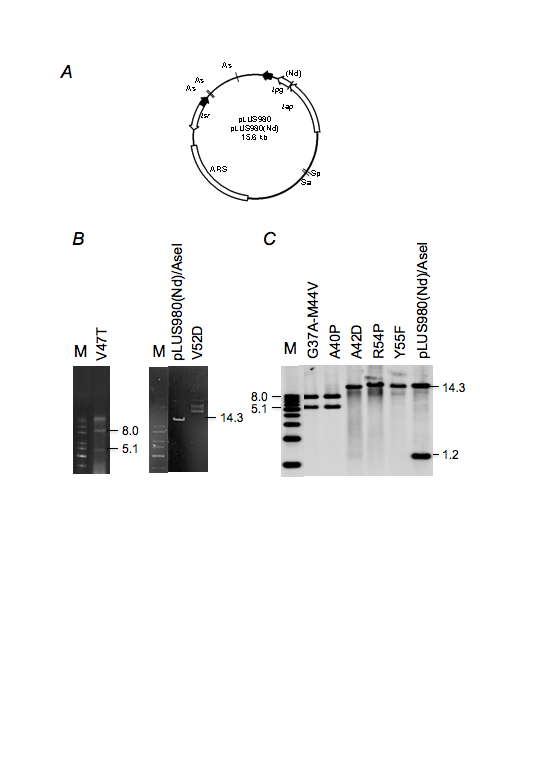

Supplement: Figure S6 — Topology of pLUS980(Nd) derivatives containing mutations in the HTH domain of tpg . (A) Physical maps of plasmids pLUS980 and pLUS980(Nd). (B) Genomic DNA isolated from MR04 transformants containing a pLUS980(Nd) derivative was digested with SacI (Sa) and electrophoresized. The mutations (V47T, V52D) in tpg are indicated. pLUS980(Nd) DNA digested by AseI (As) and 1-kb DNA ladder (‘M’) are used as size markers. (C) Genomic DNA isolated from MRO4 transformants were digested with SpeI (Sp), electrophoresized, and hybridized with pLUS980 DNA as probe. The mutations in tpg are indicated. The sizes of the circularized plasmid DNA derived from the transforming linear plasmid DNA varied from transformants to transformants, depending on the end joining sites. Shown here are representative cases. (TIFF) [file pone.0056322.s006.tif]
